# Impact of host phonons on interstitial diffusion

**DOI:** 10.1038/s41598-022-11662-2

**Published:** 2022-05-12

**Authors:** Chunguang Tang, Gang Sun, Yun Liu

**Affiliations:** 1 Research School of Chemistry, The Australian National University, Canberra, Australia; 2 Institute of Climate, Energy and Disaster Solutions, The Australian National University, Canberra, Australia; 3 Department of Fundamental Engineering, Institute of Industrial Science, University of Tokyo, 4-6-1 Komaba, Meguro-ku, Tokyo 153-8505 Japan

**Keywords:** Materials science, Physics

## Abstract

The net effect of host phonons on interstitial diffusion has remained as a fundamental knowledge gap in our current theories since the motions of the host atoms and interstitials were coupled in these theories. Here we study this effect through molecular dynamics simulations of hydrogen diffusion in palladium, in which the motions can be decoupled through pinning the host atoms. Mathematically this decoupling corresponds to expanding the total diffusion coefficient into a Taylor series, which separates the phonon contribution from the intrinsic interstitial jumping. Our results clearly show that palladium phonons significantly promote hydrogen diffusion. The phonon contribution, being linear with temperature at high temperatures and exponential at low temperatures, is fitted with Brownian motion model. The total diffusion of interstitials can be understood as the intrinsic interstitial jumping in a pinned host plus phonon-induced Brownian diffusion. The generality of our findings is validated by examining the motion of lithium in manganese oxide and carbon in iron.

Lattice vibrations or phonons play a major role in many physical properties, such as thermal and electrical conductivity, of condensed matter systems. Their effect on mass transport, i.e., atomic diffusion, however, is not well understood despite studies for decades. It is well known that the diffusion in solids occurs via atomic jumping and follows the Arrhenius equation. In particular, based on the established random walk theory^1,2^ the diffusion coefficient for interstitials can be written as

1 $$\begin{aligned} D= D_0 {\mathrm{exp}}\left( \frac{-E_a}{k_BT}\right) \end{aligned}$$

where $E_a$ is the activation enthalpy for the jump, $k_B$ is the Boltzmann constant, and $D_0$ is a constant virtually independent of temperature *T* and combines the effects of solid structures, interstitial vibration frequency, and an entropy change term associated with the jump. In the early versions^2,3^ of the random walk theory, the effect of host phonons was not considered^4^. Later, a number of classical models^4–7^ replaced the vibration frequency of the diffusing atoms with an effective frequency which involves the many body effect from the lattice phonons. The phonon effects on atomic diffusion were also discussed^8,9^ based on the quantum theory^10^. In all these theoretical studies, however, the motions of the host atoms and the jumping atom are coupled and hence the net effect or importance of phonons is not clear. In other words, although one can compute the diffusion coefficients with lattice phonons included based on these theories^11^, it is not easy to know qualitatively whether lattice vibrations positively or negatively contribute to the diffusion and quantitatively how significant the contribution is. This represents a knowledge gap for our fundamental understanding of solid diffusion, although the random walk theory has been established for decades.

The advance of our computational capacity and relevant algorithms allows us to study the above problem via simulations. Here we address the effect of lattice phonons on interstitial diffusion, using hydrogen (H) in face-centered-cubic (fcc) and amorphous palladium (Pd) as an example, based on molecular dynamics simulations. We consider the amorphous phase here because of its very different structural orders as compared to traditional crystalline solids. Hydrogen in solids has a long history in the interest of scientists^12,13^, and metal hydrides for hydrogen storage have seen extensive research activities over the past decades^14^. Pd is one of the first metals of which the capacity for H absorption were discovered^15^ and is still widely applied in the hydrogen energy sector^16,17^, for example, as effective hydrogenation catalysts, hydrogen purification filters and hydrogen storage media. We validate the generality of our findings by examining two technically important interstitial systems, namely, carbon (C) in iron (Fe) and lithium (Li) in manganese oxide ($\hbox {MnO}_2$).

**Figure 1:**
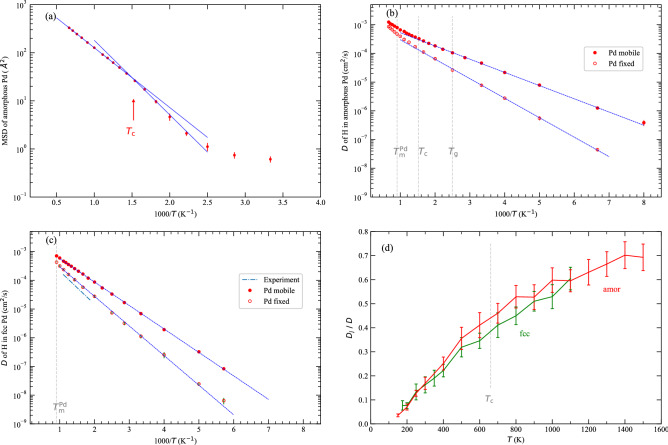
Diffusion of H in Pd matrix, with Pd fixed and not fixed, respectively. (**a**) MSD of amorphous Pd atoms with respect to temperature at a given sampling time *t* = 100 ps. $T_c$ ($\sim$ 660 K) is the estimated mode coupling temperature for Pd diffusion. (**b**) Hydrogen diffusion in amorphous Pd. The Arrhenius fitting gives $D_0$ = 1.52$\times 10^{-3}$ and $E_a$ = 0.091 (in cm$^2$/s and eV, same for below), and $D_0$ = 1.43$\times 10^{-3}$ and $E_a$ = 0.13 for Pd fixed. $T_g$ ($\sim$ 400 K) is the estimated glass transition temperature, and $T_m$ ($\sim$ 1100 K) is the melting point of fcc Pd. (**c**) Hydrogen diffusion in fcc Pd. The Arrhenius fitting gives $D_0$ = 3.76$\times 10^{-3}$ and $E_a$ = 0.16 , and $D_0$ = 3.20$\times 10^{-3}$ and $E_a$ = 0.20 if Pd is fixed. The experimental diffusion data are from reference^18^. (**d**) The ratio of H diffusion coefficient in fixed Pd ($D_j$) to that in mobile Pd (*D*). The errorbars are for standard deviation of 10 independent simulations (same for other figures unless otherwise specified). The data for H diffusion are in Supplementary Information.

To investigate the amorphous phase, a Pd-H system with 4000 Pd atoms and 200 H atoms was well liquefied at 2100 K and quenched to 300 K at 10$^{14}$ K/s, and the resulting structure was confirmed to be amorphous based on its Pd-Pd pair distribution function. For the crystalline system, we randomly put 200 H atoms at the octahedral interstitial sites of an fcc structure of 4000 Pd atoms. We relaxed the obtained crystalline structures at various temperatures to obtain the equilibrium system volume. These computations were carried out using the NPT (constant atom number, pressure, and temperature) ensemble by setting the normal pressures to zero. Using the NVT (constant atom number, volume, and temperature) ensemble at various temperatures with corresponding system volume obtained from the NPT ensemble, we then computed the mean squared displacements (MSDs) of H and Pd atoms in the obtained structures for various times after an initial relaxation for 50 ps or so. The MSD sampling time ranges from 0.1 to 200 ns, depending on temperature, Pd phase, and whether Pd atoms were pinned. The varying sampling time is to ensure the motion of H in a given situation becomes stable. Each MSD data point in this work represents the average over about ten independent computations. More details of MSD sampling can be found elsewhere^19^. The MSD data were used to compute the diffusion coefficient based on the Einstein relation

2 $$\begin{aligned} D=\frac{1}{6}\lim _{t\rightarrow \infty } \frac{\partial \langle r^2(t) \rangle }{\partial t} \end{aligned}$$

where *t* is the time and $\langle r^2(t) \rangle$ is the ensemble average of MSDs of diffusing atoms. For H diffusion, only MSD data higher than 10 Å$^2$ were used to make sure the system is in the diffusive regime. Pd diffusion is much slower, and hence we only sampled the MSD at a fixed time length (100 ps) for implicating its mobility. The computations for carbon in crystalline iron are similar to the above. The atomic interactions of the Pd-H system were described by an embedded-atom-method-based potential^20^. Simulations^21^ based on this potential has confirmed the concentration dependence of hydrogen diffusion in metals as observed experimentally. All the simulations in this work were performed using the code LAMMPS (ref.^22^) with Nos$\acute{\mathrm{e}}$-Hoover thermostat and barostat, and the timestep was set as 1 fs.

We started our study with the thermodynamics of Pd matrix. For fcc Pd, its simulated melting point was found to be $\sim$1100 K. For the amorphous phase below the glass transition temperature, $T_g$, Pd atoms essentially only vibrate about their local favorable positions within the time scale of interest to this study (Fig. 1a). Above $T_g$, the MSD of Pd increases monotonically with temperature. During the simulations, crystallization of Pd at elevated temperatures was avoided by selecting a reasonably short time scale (but long enough for observing stable H diffusion). We note the existence of a critical temperature ($T_c$) in the supercooled region, as also reported for Pd in multiple-component amorphous alloys^23^, which corresponds to the turning point of the Arrhenius curve and signifies the change in the diffusion mode from liquid-like motion to solid-like hopping upon cooling^23^. For the purpose of this work, we limit our discussion on H diffusion in the amorphous phase below $T_c$.

We study the net effect of host phonons by comparing H diffusion with the vibrations of Pd atoms enabled and disabled, respectively. While impossible in experiments, this strategy is convenient in simulations and useful for tackling complex problems^24^. We first simulated the diffusion of H atoms with the Pd atoms fixed (by setting the force on them to zero) after the system was annealed for some time. Note that in this case, the kinetic energy of H is still connected to an external thermostat in simulations. From a theoretical perspective, in this case the collision between H and Pd is elastic since the collision does not transfer kinetic energy to Pd. As indicated by the open circles in Fig. 1b–c, H diffusion in this case fits well into the Arrhenius equation. As can be seen, like the random walk theory, our simulations based on the static Pd matrices predict a well-behaved Arrhenius diffusion of H. We note that in the random walk theory lattice relaxation during the jumping of interstitials is possible. Here the host matrix is relaxed for some random time before it is fixed.

Next we computed *D* of H without pinning the Pd atoms. As indicated by the solid circles in Fig. 1b–c, H diffusion in this scenario also follows the Arrhenius relationship. Figure 1c also shows the experimental^18^ *D* of hydrogen from $\sim 530$ to $\sim 910$ K, which differs from our calculations within one order of magnitude. Other measurements^25,26^ reported the values of *D* near 300 K to be $\sim 1.6-1.9\times 10^{-7}$ cm$^2$/s, which are close to the extrapolation of the previous measurement^18^ and differ from our calculations by more than one order of magnitude. Such a difference mainly results from the higher activation energy reported by experiments, which is not surprising in view of the trapping of hydrogen by lattice defects in experiments^27^. The accuracy of the EAM potential used in this work may also contribute to the difference, but overall the calculations resemble the trend of experiments.

Figure 1b–c clearly shows a general and significant difference in the diffusion coefficients for fixed and mobile Pd. We label the diffusion coefficients for the case of fixed Pd as $D_j$ since here only H jumping effect contributes to the diffusion. Figure 1d shows that the ratio $D_j/D$ decreases as temperature decreases and reaches below 30% at room temperature. To investigate the effect of Pd phonons on H diffusion, we split the total diffusion coefficient *D* as

3 $$\begin{aligned} D=D_j+D_{ph} \end{aligned}$$

where $D_j$ by itself has the Arrhenius form $D_j=D_0^j {\mathrm{exp}}(-E_a^j/RT)$, and $D_{ph}$ represents the contribution of Pd phonons. The philosophy behind this treatment is as follows. The Pd phonon effect is related to the mass of Pd ($m_{{\mathrm{Pd}}}$). Imagine H diffuses in a hypothetical Pd isotope with infinite mass ($w=1/m_{{\mathrm{Pd}}}=0$). In this case, the vibration amplitude of Pd becomes zero and *D* reduces to $D_j$. As *w* increases, the phonon effect emerges and contributes to H diffusion. This led us to formally write *D* as a Taylor series

4 $$\begin{aligned} D(w)=D(0)+D'(0)w+\frac{D''(0)}{2}w^2+\frac{D'''(0)}{3!}w^3+\cdots \end{aligned}$$

where $D(0)=D_j$ and the rest items can be wrapped as $D_{ph}$ representing the phonon effect.

In the following we study $D_{ph}$ from the perspective of Brownian motion. Brownian motion is generally regarded as a fluid phenomenon, but similar phenomena, such as thermal noise in electric conductors^28^, exist in solids and all of them follow the fluctuation-dissipation theorem^29^. The splitting of *D* may raise concerns since the Brownian motion model is space/time continuous while the interstitials move in a space-discrete force field determined by the host structure. We note equation 3 implicitly treats the force field created by the host atoms as

5 $$\begin{aligned} \varepsilon =\varepsilon _f +\varepsilon _v \end{aligned}$$

where $\varepsilon _f$ corresponds to the host atoms at their pinned positions and the fluctuating augmentation $\varepsilon _v$ arises from host phonons. The $\varepsilon _f$ field results in a discrete diffusion component ($D_j$) and the Brownian contribution comes only from $\varepsilon _v$ which is continuous in space/time. Such a treatment is indeed consistent with the solid-liquid phase transition: as temperature increases, the $\varepsilon _v$ component increases and eventually the force field becomes liquid-like.

**Figure 2:**
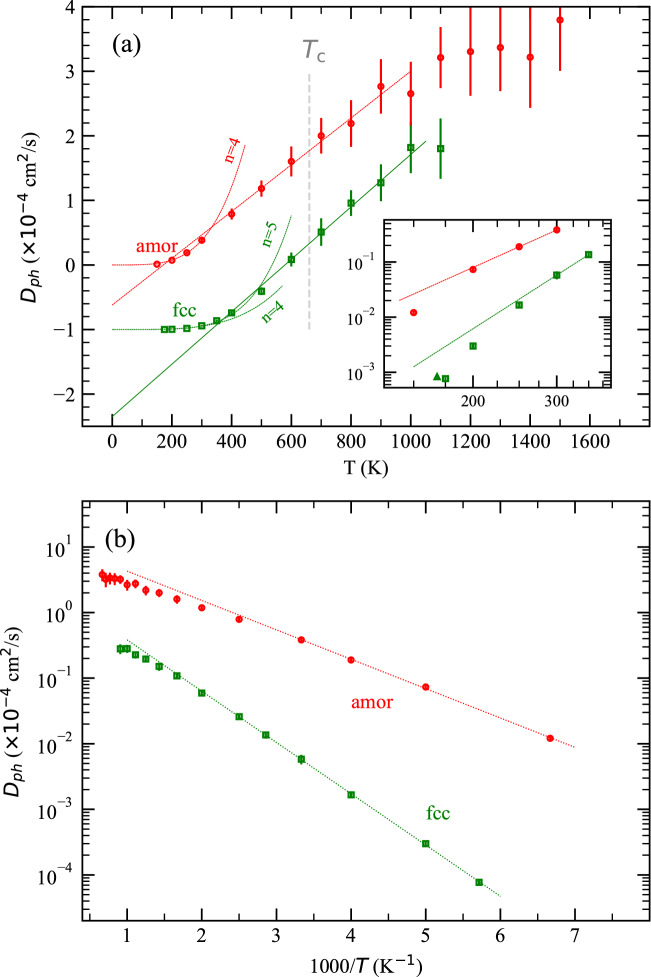
Contribution of Pd phonons to H interstitial diffusion. The data points for the amorphous phase above $T_c$ are shown only for reference. (**a**) The linear parts can be fitted with $D_{ph}$ = (4.06 $\times \,10^{-3}T-1.35$)$\times \,10^{-4}$ cm$^2$/s and (3.62 $\times \,10^{-3}T-0.62$)$\,\times \,10^{-4}$ cm$^2$/s for the fcc and amorphous phases, respectively. Power functions $D_{ph}$ = 6.42$T^4\,\times \,10^{-16}$ cm$^2$/s and 2.27$T^5\,\times \,10^{-18}$ cm$^2$/s were used to fit the fcc phase, and $D_{ph}$ = 4.74$T^4\,\times \,10^{-15}$ cm$^2$/s was used for the amorphous phase. The plotted data for the fcc phase are shifted downwards for clarity. Inset: Zoom-in for low temperatures in log scales, same units as in the main. The linear lines are for eye guide only. The triangle indicates the upper limit of $D_{ph}$ (by setting $D_j$ = 0) of fcc at 175 K (triangle slightly shifted to the left for clarity). (**b**) Arrhenius plot of $D_{ph}$.

The diffusion coefficient of Brownian particles suspended in a fluid can be written as (see reference^30,31^ or the Supplementary Information)

6 $$\begin{aligned} D_{B}=\frac{{\overline{E}}_{k}}{9\pi \mu a} \end{aligned}$$

where ${\overline{E}}_k$ and *a* are the average kinetic energy and size of the Brownian particles, respectively, and $\mu$ is the liquid viscosity. A more familiar form for equation (6) is $D_B=k_BT/6\pi \mu a$ since ${\overline{E}}_{k}$ equals $3k_BT/2$ based on the equipartition theorem. In view that H atoms are in thermal equilibrium with Pd phonons, we can directly apply Equation (6) to $D_{ph}$ if we know the average energy of Pd phonons ${\overline{E}}_{ph}$. In contrast to liquid of which $\mu$ is sensitive to temperature, here we can assume constant $\mu$ for Pd phonons. In other words, we assume a constant friction coefficient for the motion of H in the phonon fluid. At low temperatures^32,33^, ${\overline{E}}_{ph}$ is approximately proportional to $T^n$, where *n* is some constant. Substituting this into ${\overline{E}}_{k}=3{\overline{E}}_{ph}/2$, we can write

7 $$\begin{aligned} D_{ph}\propto T^n \,\,\,\,\,\,\,\,\,\,\,\,\, ({\mathrm{For\,low}}\,T) \end{aligned}$$

At high temperatures, the equipartition theorem gives ${\overline{E}}_{ph}\approx k_BT$. Note the actual ${\overline{E}}_{ph}$ (=$\int _0^T CdT$) is smaller than $k_BT$ by some roughly constant number because the heat capacity per phonon, *C*, at low temperatures is lower than its high temperature limit $k_B$. In this case, we can write the phonon contribution as

8 $$\begin{aligned} D_{ph}=\frac{k_BT}{6\pi \mu a} + c \,\,\,\,\,\, ({\mathrm{For\,high}}\,T) \end{aligned}$$

where *c* is a negative constant dependent on the material.

As shown in Fig. 2a, at high temperatures $D_{ph}$ of both the amorphous and the fcc phases follows a linear behaviour with a negative intercept at $T=0$. We note that for the amorphous phase the linearity holds for a range of temperatures above $T_c$, which we attribute to the cancellation effect as at $T>T_c$ both $D_j$ and *D* deviate from the Arrhenius relationship for solids. At low temperatures, a function proportional to $T^n$ (*n* = 4 for amorphous and 5 for fcc) seems to fit $D_{ph}$ well. The fitting parameters *n* = 4 and 5 are based on the Debye approximation^32^ and the finite temperature field theory^33^, respectively. However, as shown in the log-scale inset of Fig. 2a, a constant *n* actually does not fit well with the data, which contrasts with the established models. To build a theory for this observation is beyond the scope of this work, but we note the results are logically not surprising since, if *n* can increase from 1 at high temperatures to 4 or 5 at low temperatures, it is reasonable to assume higher *n* at even lower temperatures. Inspired by this, we found the $D_{ph}$ data at low temperatures fit well with an exponential function, as shown in Fig. 2b. We note that $D_{ph}=D-D_j$ can be approximated as D as $T\rightarrow 0$ because, with a higher activation energy, $D_j$ is an infinitesimal of higher order than *D*. This explains the exponential fitting of $D_{ph}$ from the mathematical perspective.

**Figure 3:**
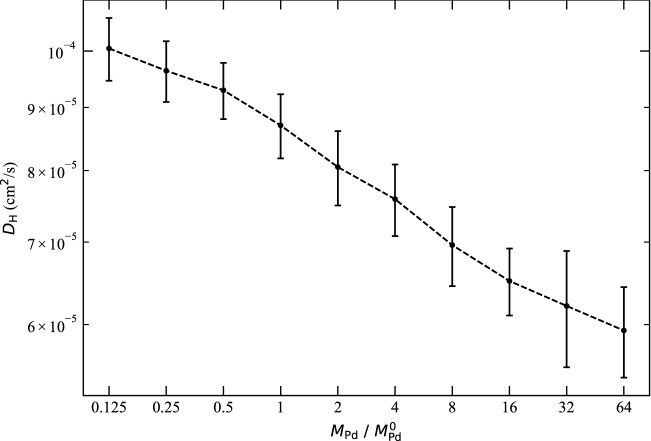
Calculated hydrogen diffusion coefficients in fcc Pd lattice at 500 K as a function of the ratio of a hypothetical Pd mass ($M_{{\mathrm{Pd}}}$) to the true Pd mass ($M^0_{{\mathrm{Pd}}}$).

For some comments to the above results, firstly we ignored the zero point vibrations in the above derivations, which only adds a small constant $D_{ph}$ that is important as *T* approaches zero. Secondly, the quantum tunneling effect for H diffusion^10^, which is important for temperatures below 200 K^34^, was not included in this work. Thirdly, we expect the phonon contribution is a general effect and hence also affect other interstitials. Indeed, a recent molecular dynamics study showed that the permeability of He atoms and $\hbox {H}_2$ molecules in amorphous silica decreases if the thermal motion of silica is forbidden^35^. To extend our findings to larger atoms, we studied the movement of carbon in fcc iron at 1500 K and that of Li$^{+}$ ions in $\hbox {Li}_{0.5} \hbox {Mn}_2 \hbox {O}_4$ at 1000 K, and the results (in Supplementary Information) confirmed the impacts of host phonons on interstitial motion. Finally, although atom-pinning decouples the motions of the host and interstitial atoms, it is a theoretical approach without an experimental counterpart. Hence, we also examined the phonon effects by only changing the mass of Pd atoms (without pinning Pd). This is equivalent to using Pd isotopes in experiments although in simulations one can arbitrarily set the mass of Pd. We used the fcc phase at 500 K as an example. Figure 3 shows that the diffusion coefficient of H decreases as the mass of Pd increases (or, equivalently, the phonon energy decreases), which supports our findings based on Pd-pinning.

In summary, based on molecular dynamics simulations we studied the effects of host phonons on interstitial diffusion, using hydrogen (H) within face centered cubic and amorphous palladium (Pd) as an example. Compared previous theoretical studies that coupled the phonons with the interstitial motions, this work decouples the two by pinning the host atoms and hence clearly reveals the net effects of host phonons. It was found that Pd phonons significantly promote H diffusion by causing Brownian-like diffusion of H atoms and dominate H diffusion below $T_{m}$/2 ($T_m$ being the melting point of Pd). Similar effects were also found for other important interstitials, such as lithium in manganese oxide and carbon in iron. This work establishes a new and improved physical picture for the general diffusion of interstitial atoms in solids and provides new perspectives for us to understand and design diffusion-related material behaviours. For example, the relaxation in metallic glasses^36^ was found to correlate with the diffusion of the smallest constituting atoms within some loosely packed/bonded regions. Such diffusion was hypothesized to relate with the vibrations of atomic strings nearby^36^, which is consistent with the current work. It is worth noting that the Brownian motion of nanosized liquid lead (Pb) inclusions within a solid aluminium (Al) was reported^37^. Although the authors found the motion is controlled by the shape of the inclusions and the diffusion of Al along the liquid/solid interface, the agitation force resulting from Al phonons seems to be an important reason for the observed motion.

## Supplementary Information

Supplementary Information.
