# Supplementary material for: Impact of host phonons on interstitial diffusion
Source: Sci Rep. 2022 May 12;12:7840. doi: 10.1038/s41598-022-11662-2 (PMC9098489; doi:10.1038/s41598-022-11662-2)
Supplement: Supplementary file 1 — Supplementary Information. [file 41598_2022_11662_MOESM1_ESM.pdf]

Impact of host phonons on interstitial diffusion

Chunguang Tang[1,2,*] Gang Sun[3], and Yun Liu[1,2]
[1] *Research School of Chemistry, The Australian National University, Canberra, Australia;* [2] *Institute of Climate, Energy and Disaster Solutions, The Australian National University, Canberra, Australia;* [3] *Department of Fundamental Engineering, Institute of Industrial Science, University of Tokyo, 4-6-1 Komaba, Meguro-ku, Tokyo 153-8505, Japan.*

This Supplementary Information includes Figures 1-2, Table I and discussion of the following points:
1. Structures of amorphous Pd matrix;
2. Langevin's derivation of the Brownian diffusion coefficient
3. H diffusion data in this study.
4. Host phonon effects on carbon and lithium interstitials

1. Structures of amorphous Pd matrix

Fig. 1 shows the Pd-Pd pair distribution function $g(r)$ of the Pd-H solids annealed at various temperatures. For the amorphous phase, the figure indicates that as temperature increases the structure changes from amorphous solid into liquid. The splitting of the second $g(r)$ peak[6,7] is characteristic of the amorphous structure. In Fig. 1, the position of the first peak represents the mean distance between neighbor Pd atoms, and the half width of the first peak indicates the mean vibration amplitude of a Pd atom. It is clear from the figure that the vibration amplitude positively correlates with temperature.

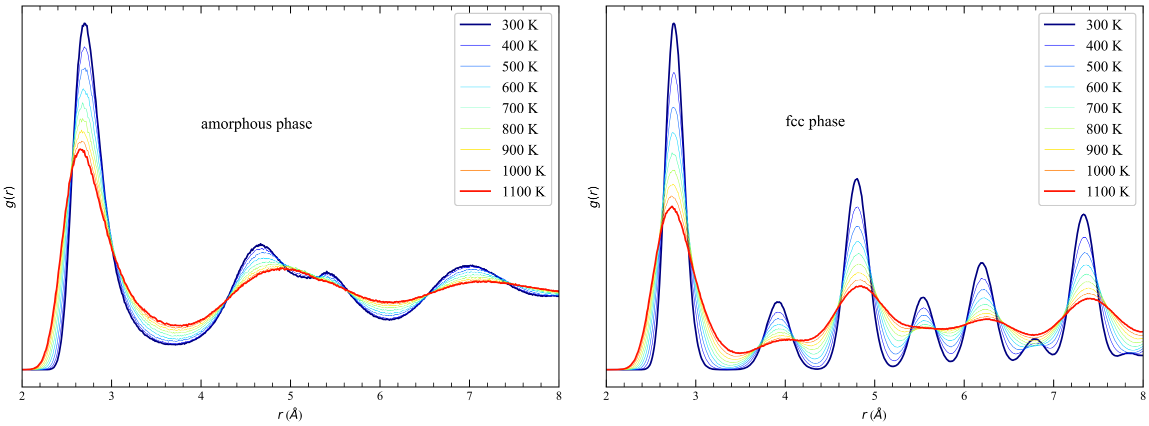

FIG. 1. Pd-Pd pair distribution function $g(r)$ of the amorphous and fcc Pd-H solid annealed at various temperatures.

2. Langevin's derivation of the Brownian diffusion coefficient

In his original paper, Langevin described the motion of a particle in the direction x within a liquid as

$$m\frac{dv_x}{dt} = -6\pi\mu a v_x + X \tag{1}$$

where m, a, and v_x are the mass, radius, and speed of the particle, respectively, μ is the viscosity of the liquid, and X is a complementary force that 'is indifferently positive and negative' and 'maintains the agitation of the particle'. At temperature T, the particle is in thermal equilibrium with the liquid molecules and has the average kinetic energy in the direction x as $\overline{E}_{k,x} = k_B T/2$. The solution of Equation 1 gives the average MSD in the direction x for a time interval τ as $\overline{\Delta_x^2} = 2\overline{E}_{k,x}\tau/(3\pi\mu a)$. Extending the solution to 3D and using Einstein relation $D = \lim_{t\to\infty} \partial\langle r^2(t)\rangle/(6\partial t)$ one

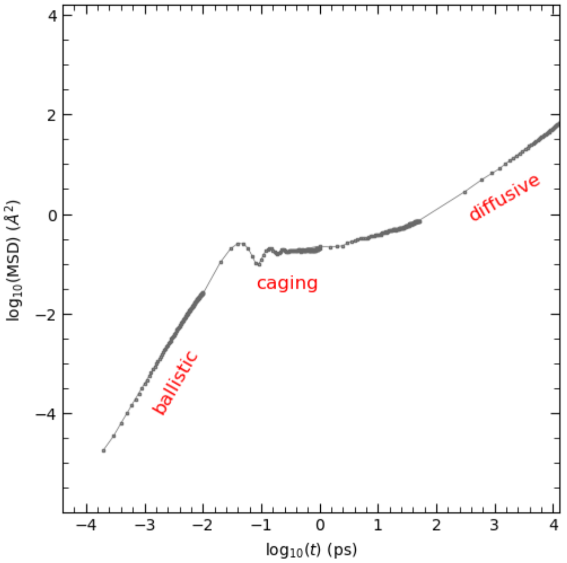

FIG. 2. Example MSD of H versus time in log scale for the fcc phase at 175 K. Independent computations for several different time scales were carried out to show the whole-scale behaviour. Slight offsets among these independent data sets were removed by uniformly shifting the MSD data along the vertical axis.

obtains the diffusion coefficient of a Brownian particle as

$$D_B = \frac{\overline{E}_k}{9\pi\mu a} \tag{2}$$

A more familiar form for D_B is $k_B T/6\pi\mu a$, which illustrates the dependence of Brownian motion on T. Note μ for fluids also depends on T.

3. H diffusion data in this study

The mean squared displacement (MSD) of atoms as a function of time can be divided into three regimes[8]. When time is very short, the diffusion is in the "ballistic" regime where atoms move without colliding with other atoms, and MSD is proportional to t^2 where t is time. The second is the "caging" regime where atoms collide with their neighbours and move forth and back. In this regime, MSD is roughly a constant as time increases. The timescale for the caging regime is temperature and system dependent. Bebyond the caging regime is the "diffusive" regime where MSD is proportional to t and the Einstein relation $D = \frac{1}{6}\lim_{t\to\infty}\frac{\partial\langle r^2(t)\rangle}{\partial t}$ applies. For example, these regimes are shown in Fig. 2 for the fcc phase (Pd atoms not fixed) at 175 K. In the log scales, the ballistic regime has a slope of 2 and the deep diffusive regime has a slope of 1.

It is important to make sure that the sampled MSDs are within the diffusive regime. As shown in Table I, all the diffusion data in this study are within the diffusive regime except the case of hydrogen diffusion in fixed fcc Pd at 175 K, which is in a relatively shallow diffusive regime. This case has a slope of ~0.7, which could result in a slightly overestimated diffusion coefficient. However, we do not expect this single case to alter our conclusions on the phonon contribution to hydrogen diffusion since, as mentioned in the main text, at such low temperatures the contribution of hydrogen hopping is significantly smaller than that of phonons and hence is negligible.

4. Host phonon effects on carbon and lithium interstitials

Hydrogen is the lightest element. To confirm the findings in this work also hold for other larger/heavier interstitials, we performed similar calculations for the motion of carbon in iron and lithium ion in spinel cathode material $Li_{0.5}Mn_2O_4$. The former system contains 200 C and 4000 Fe atoms, and a many-body Tersoff potential[9] was used for atomic interactions. The latter system contains 256 Li^+, 256 Mn^{3+}, 768 Mn^{4+} and 2048 O^{2-} ions, which was obtained by randomly removing 50% Li ions from $LiMn_2O_4$ and switching some Mn^{3+} to Mn^{4+} for charge neutrality,

TABLE I. Hydrogen diffusion data in amorphous (top) and fcc (bottom) Pd. Column 'MSD' gives the lower and (averaged) upper limits of MSD (in unit Å^2) used for diffusion coefficient fitting, and the fitted diffusion coefficient and its standard deviation are shown in columns 'D' and 'STD', in units cm^2/s. Column 'slope' corresponds the log slopes of the MSD data as in Fig. 2, and column 't' indicates the MSD sampling time (in unit ns) needed for reaching the upper limit of MSD.

T (K)	Pd-mobile					Pd-fixed				
	MSD	slope	D	STD	t	MSD	slope	D	STD	t
amorphous										
125	200, ∼250	0.94	3.87e-07	9.28e-08	10					
150	300, ∼800	0.89	1.26e-06	1.42e-07	10	300, ∼570	0.91	4.44e-08	5.84e-09	200
200	100, ∼500	0.92	7.88e-06	5.90e-07	1	300, ∼700	0.92	5.53e-07	8.87e-08	20
250	100, ∼660	0.94	2.17e-05	1.60e-06	0.5	100, >1000	0.95	2.76e-06	1.72e-07	10
300	100, ∼280	0.98	4.61e-05	3.93e-06	0.1	200, ∼470	0.97	7.77e-06	9.51e-07	1
350	100, ∼440	0.99	7.12e-05	6.35e-06	0.1					
400	100, ∼640	1.00	1.05e-04	8.31e-06	0.1	100, >1000	0.94	2.64e-05	1.92e-06	1
450	100, ∼820	0.98	1.40e-04	1.19e-05	0.1					
500	100, >1000	1.00	1.84e-04	9.78e-06	0.1	200, ∼400	0.95	6.54e-05	7.75e-06	0.1
550	100, >1000	0.98	2.24e-04	1.16e-05	0.1					
600	100, >1000	1.02	2.72e-04	2.01e-05	0.1	100, ∼690	0.93	1.12e-04	1.15e-05	0.1
650	100, >1000	1.01	3.25e-04	2.41e-05	0.1					
700	100, >1000	1.01	3.71e-04	2.59e-05	0.1	100, >1000	0.96	1.71e-04	9.40e-06	0.1
750	100, >1000	1.02	4.15e-04	2.77e-05	0.1					
800	100, >1000	1.00	4.65e-04	3.40e-05	0.1	100, >1000	0.98	2.46e-04	1.23e-05	0.1
850	100, >1000	1.00	5.10e-04	4.05e-05	0.1					
900	100, >1000	1.02	5.85e-04	3.47e-05	0.1	100, >1000	0.97	3.08e-04	2.39e-05	0.1
1000	100, >1000	1.00	6.60e-04	4.49e-05	0.1	100, >1000	0.98	3.95e-04	2.04e-05	0.1
1100	100, >1000	1.02	7.94e-04	3.77e-05	0.1	100, >1000	0.98	4.73e-04	2.87e-05	0.1
1200	100, >1000	1.01	8.95e-04	6.31e-05	0.1	100, >1000	0.99	5.64e-04	2.59e-05	0.1
1300	100, >1000	1.01	1.00e-03	5.85e-05	0.1	100, >1000	1.00	6.68e-04	3.36e-05	0.1
1400	100, >1000	1.02	1.08e-03	7.08e-05	0.1	100, >1000	1.00	7.57e-04	3.41e-05	0.1
1500	100, >1000	1.02	1.24e-03	5.54e-05	0.1	100, >1000	1.00	8.56e-04	5.62e-05	0.1
fcc										
175	30, ∼150	0.95	8.34e-08	4.31e-09	30	10, ∼25	0.67	6.43e-9	1.59e-9	50
200	10, ∼200	0.99	3.24e-07	2.72e-08	10	70, ∼160	0.90	2.45e-08	3.43e-09	100
250	10, ∼120	0.96	1.92e-06	1.51e-07	1	40, ∼80	0.93	2.55e-07	6.00e-08	5
300	10, ∼40	0.94	6.94e-06	9.40e-07	0.1	20, ∼70	0.95	1.13e-06	1.75e-07	1
350	10, ∼100	0.99	1.68e-05	1.55e-06	0.1	40, ∼200	0.93	3.20e-06	4.71e-07	1
400	10, ∼200	1.00	3.32e-05	2.57e-06	0.1	40, ∼450	0.91	7.38e-06	6.60e-07	1
450	10, ∼330	0.98	5.44e-05	3.10e-06	0.1					
500	10, ∼520	1.00	8.70e-05	5.21e-06	0.1	40, ∼170	0.94	2.78e-05	3.06e-06	0.1
550	10, ∼730	1.01	1.19e-04	7.18e-06	0.1					
600	10, ∼970	1.02	1.66e-04	1.01e-05	0.1	10, ∼360	0.91	5.73e-05	3.91e-06	0.1
650	10, >1000	1.03	2.07e-04	1.62e-05	0.1					
700	10, >1000	1.02	2.56e-04	1.84e-05	0.1	10, ∼640	0.95	1.05e-04	1.08e-05	0.1
750	10, >1000	1.03	3.10e-04	1.55e-05	0.1					
800	10, >1000	1.02	3.56e-04	1.68e-05	0.1	10, ∼980	0.95	1.60e-04	1.07e-05	0.1
850	10, >1000	1.01	4.16e-04	2.57e-05	0.1					
900	10, >1000	1.02	4.64e-04	2.48e-05	0.1	10, >1000	0.97	2.37e-04	1.41e-05	0.1
1000	10, >1000	1.04	5.99e-04	3.09e-05	0.1	10, >1000	0.98	3.17e-04	2.50e-05	0.1
1100	10, >1000	1.04	7.08e-04	4.09e-05	0.1	10, >1000	1.01	4.28e-04	2.25e-5	0.1

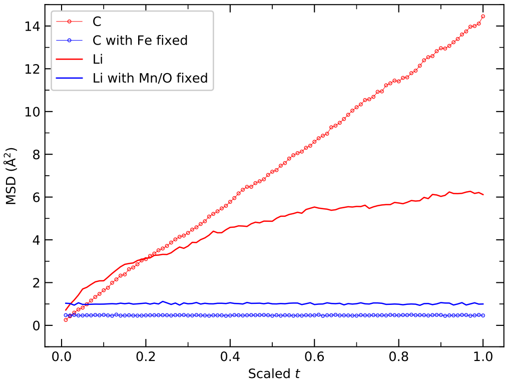

FIG. 3. Mean squared displacements of C in γ-Fe at 1500 K and Li in $Li_{0.5}Mn_2O_4$ at 1000 K, with the host atoms fixed or not. The scaled time t=1 represents 1 ns for Li and 10 ns for C, respectively. Data averaged over five independent calculations. The data for the fixed hosts are amplified by 10 times for clarity, and their near constant values indicate C and Li are caged by their neighbor particles within the time range.

and a Gilbert-Ida-type pair potential[10] was used for the system. The potential contains a constant parameter f_0, which was optimized in this work such that the calculated lattice constants agree with the experimental ones. For $Li_{0.5}Mn_2O_4$, the Ewald method was used for computing the long-range coulombic interactions. Fig. 3 confirmed the impacts of host phonons on interstitial motion.

---

* chunguang.tang@anu.edu.au
[6] B. Vandewaal, J. Non-Cryst. Solids **189**, 118 (1995), wOS:A1995RR67300013 bibtex: vandewaal_origin_1995.
[7] S. P. Pan, J. Y. Qin, W. M. Wang, and T. K. Gu, Phys. Rev. B **84**, 092201 (2011), bibtex: pan_origin_2011.
[8] C. Donati, S. C. Glotzer, P. H. Poole, W. Kob, and S. J. Plimpton, Phys. Rev. E **60**, 3107 (1999), place: College Pk Publisher: American Physical Soc WOS:000082778200087.
[9] K. O. E. Henriksson, C. Bjorkas, and K. Nordlund, J. Phys.-Condes. Matter **25**, 445401 (2013), place: Bristol Publisher: Iop Publishing Ltd WOS:000325897900017.
[10] K. Suzuki, Y. Oumi, S. Takami, M. Kubo, A. Miyamoto, M. Kikuchi, N. Yamazaki, and M. Mita, Jpn. J. Appl. Phys. **39**, 4318 (2000), place: Minato-Ku Tokyo Publisher: Japan J Applied Physics WOS:000088910000017.

---

* chunguang.tang@anu.edu.au
[1] Vandewaal B, On the Origin of 2nd-Peak Splitting in the Static Structure Factor of Metallic Glasses, *J. Non-Cryst. Solids*, **189**, 118, (1995).
[2] Pan SP, Qin JY, Wang WM, and Gu TK, Origin of splitting of the second peak in the pair-distribution function for metallic glasses, *Phys. Rev. B*, **84**, 092201, (2011).
[3] Donati C, Glotzer SC, Poole PH, Kob W, and Plimpton SJ, Spatial correlations of mobility and immobility in a glass-forming Lennard-Jones liquid, *Phys. Rev. E*, **60**, 3107, (1999).
[4] Henriksson KOE, Bjorkas C, and Nordlund K, Atomistic simulations of stainless steels: a many-body potential for the Fe-Cr-C system, *J. Phys.-Condes. Matter*, **25**, 445401, (2013).
[5] Suzuki K *et al.*, Structural properties of LixMn2O4 as investigated by molecular dynamics and density functional theory, *Jpn. J. Appl. Phys.*, **39**, 4318, (2000).
[6] B. Vandewaal, J. Non-Cryst. Solids **189**, 118 (1995), wOS:A1995RR67300013 bibtex: vandewaal_origin_1995.
[7] S. P. Pan, J. Y. Qin, W. M. Wang, and T. K. Gu, Phys. Rev. B **84**, 092201 (2011), bibtex: pan_origin_2011.
[8] C. Donati, S. C. Glotzer, P. H. Poole, W. Kob, and S. J. Plimpton, Phys. Rev. E **60**, 3107 (1999), place: College Pk Publisher: American Physical Soc WOS:000082778200087.
[9] K. O. E. Henriksson, C. Bjorkas, and K. Nordlund, J. Phys.-Condes. Matter **25**, 445401 (2013), place: Bristol Publisher: Iop Publishing Ltd WOS:000325897900017.
[10] K. Suzuki, Y. Oumi, S. Takami, M. Kubo, A. Miyamoto, M. Kikuchi, N. Yamazaki, and M. Mita, Jpn. J. Appl. Phys. **39**, 4318 (2000), place: Minato-Ku Tokyo Publisher: Japan J Applied Physics WOS:000088910000017
